# Jujuboside A induces bladder cancer cell apoptosis by inhibiting ATP1A2-mediated mitochondrial energy metabolism regulation

**DOI:** 10.1080/15384047.2026.2615418

**Published:** 2026-01-14

**Authors:** Meng Zhu, Yuepeng Liu, Yumin Jia, Lixin Ren, Shuhui An, Yaxuan Wang

**Affiliations:** a Department of Urology, The Second Hospital of Hebei Medical University, Shijiazhuang, China; b Department of General Practice, The Second Hospital of Hebei Medical University, Shijiazhuang, China

**Keywords:** Jujuboside A, bladder cancer cells, ATP1A2, mitochondrial energy metabolism, apoptosis

## Abstract

**Background:**

Conventional treatments for bladder cancer exhibit various limitations. Therefore, natural products, such as jujuboside A (JuA), have been explored for their multi-target effects and low toxicity. However, the specific effects of JuA in bladder cancer remain unclear.

**Objective:**

To determine whether JuA affects mitochondrial energy metabolism and apoptosis in bladder cancer cells by regulating the ATPase Na+/K+ transporting subunit alpha 2 (ATP1A2) expression.

**Methods:**

Differentially expressed genes (DEGs) in bladder cancer were analyzed using the GSE133624 dataset. ATP1A2 overexpression and knockdown bladder cancer cell models were constructed. Cell phenotypes and markers related to apoptosis and mitochondrial energy metabolism were assessed. Moreover, targeting effects of JuA were investigated.

**Results:**

Interleukin (IL)-6, ATP1A2, and hydroxysteroid 11-beta dehydrogenase 1 were identified as potential JuA targets, with ATP1A2 being the main target. ATP1A2 overexpression enhanced the viability and inhibited the apoptosis of bladder cancer cells and promoted mitochondrial energy metabolism in vitro, whereas ATP1A2 knockdown had the opposite effects. JuA decreased cell viability, inhibited ATP1A2 expression, and disrupted mitochondrial energy metabolism. These anticancer effects of JuA were reversed by ATP1A2 overexpression.

**Conclusion:**

This study elucidated the molecular mechanism by which JuA regulates mitochondrial energy metabolism and induces apoptosis in bladder cancer cells through targeted inhibition of ATP1A2. These findings reveal the crucial role of ATP1A2 in the energy metabolism and survival of bladder cancer cells, providing a new molecular perspective for a deeper understanding of the pathological mechanisms of bladder cancer.

## Introduction

Bladder cancer accounts for 80%–90% of urothelial carcinoma cases. Painless hematuria is the primary early symptom of bladder cancer. However, owing to its strong concealment, approximately 10%–15% of patients already exhibit metastasis at initial diagnosis, with a postoperative recurrence rate as high as 50%.^1^ Radical cystectomy is the main treatment option for early-stage bladder cancer; however, the postoperative recurrence risk remains high.^2^ Advanced-stage patients rely on platinum-containing chemotherapy or immune checkpoint inhibitors, such as programmed cell death-1/programmed cell death-ligand 1 inhibitors. However, chemotherapy is associated with severe toxicity and high drug resistance.^3^ Antibody–drug conjugates, such as enfortumab vedotin combined with pembrolizumab, have significantly improved patient survival (median overall survival [OS] = up to 31.5 months). However, their use is limited by various challenges, such as strong target dependence, adverse reactions, and high treatment costs.^4^^,^^5^ To overcome these treatment bottlenecks, natural products have been explored for new antitumor drugs with multi-target regulatory effects and low toxicity.^6^ This study focused on one such natural product, jujuboside A (JuA), for several reasons. First, this compound exhibits anti-inflammatory, antioxidant, and apoptosis-inducing activities against tumor cells.^7-9^ Second, owing to its natural origin, it does not exert the toxic side effects of traditional chemotherapy and enhances treatment efficacy by regulating the key signaling pathway, the Hippo pathway, and inhibiting YAP nuclear translocation.^10^ Our study aimed to provide safe and effective treatment options for the precision treatment of bladder cancer.

As the α2 subunit of sodium-potassium adenosine triphosphatase (Na^+^/K^+^-ATPase), ATP1A2 plays crucial roles in maintaining the ion gradient across the cell membrane and signal transduction. Its abnormal expression is closely associated with the occurrence of various diseases and conditions, such as epilepsy and cancer.^11-13^ ATP1A2 inhibits the proliferation, invasion, migration, and epithelial–mesenchymal transition of prostate cancer cells by regulating the transforming growth factor-β/Smad signaling pathway, which exerts tumor-suppressing effects.^14^ Mitochondria, the cellular energy metabolism center, are possibly involved in ATP1A2 regulation. ATP1A2 affects the mitochondrial functions and energy metabolism by regulating glutamate uptake.^15^ Reactive oxygen species (ROS) generation and calcium ion homeostasis regulation make mitochondria key hubs connecting energy metabolism and apoptosis.^16^ Mitochondrial dysfunction impairs the electron transport chain and leads to excessive ROS accumulation and cytochrome c release, thereby activating the caspase cascade and triggering the intrinsic apoptotic pathway.^17^ Therefore, exploration of the interactions among ATP1A2, mitochondrial energy metabolism, and apoptosis will elucidate the molecular mechanisms underlying mitochondrial energy metabolism imbalance in bladder cancer and provide a theoretical basis for the development of targeted treatment strategies for this disease.

In this study, differentially expressed genes (DEGs) in bladder cancer were detected using the GSE138125 dataset. By assessing the JuA target genes and gene overlaps with the DEGs, three candidate genes (interleukin [*IL*]-*6*, ATPase Na+/K+ transporting subunit alpha 2 [*ATP1A2*], and hydroxysteroid 11-beta dehydrogenase 1 [*HSD11B1*]) were identified. We selected ATP1A2 for further analyses. In vitro experiments were conducted to construct ATP1A2 overexpression and knockdown cell lines. Phenotypic assays, such as proliferation and apoptosis assays, were performed to verify the cancer-promoting effects of ATP1A2 in bladder cancer. Subsequently, JuA was used to treat bladder cancer cells, and changes in ATP1A2 protein levels and mitochondrial energy metabolism were examined. Notably, JuA targeted and inhibited ATP1A2 protein expression. This study elucidates the carcinogenic mechanisms of ATP1A2 in bladder cancer and provides an experimental basis for the development of precise ATP1A2-targeted treatment strategies targeting ATP1A2.

## Results

### Screening for potential JuA targets among the DEGs detected in bladder cancer

**Figure 1.**
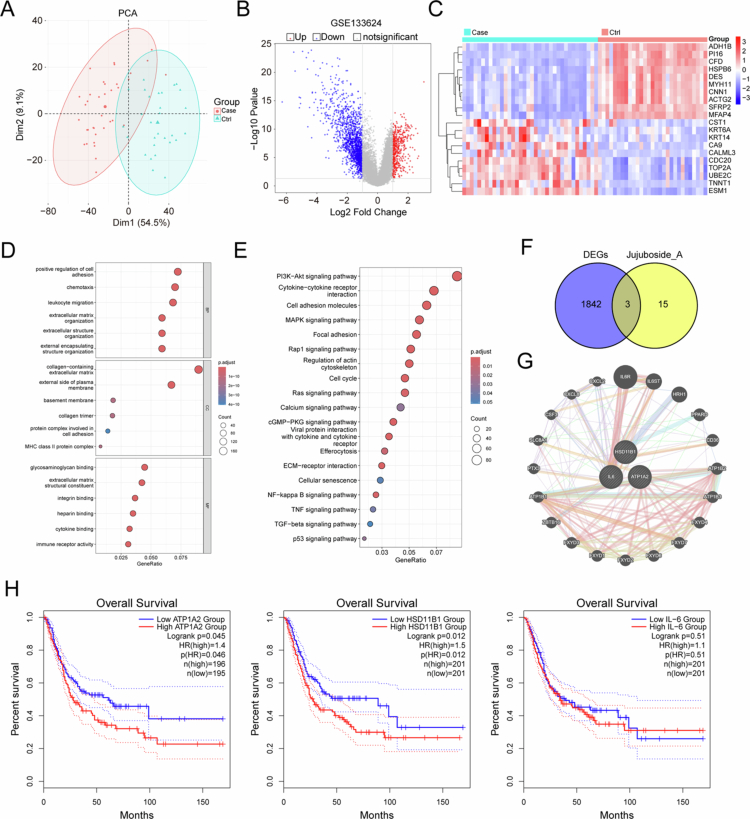
Screening for potential jujuboside A (JuA) targets among the differentially expressed genes (DEGs) in bladder cancer. (A) Principal component analysis (PCA) of the GSE133624 dataset. (B) Volcano plots of DEGs in the GSE133624 dataset. (C) Cluster heatmap of DEGs in the GSE133624 dataset. (D) Gene ontology (GO) functional enrichment analysis of DEGs. (E) Kyoto encyclopedia of genes and genomes (KEGG) functional enrichment analysis of DEGs. (F) Venn diagram showing the overlap between DEGs in the GSE133624 dataset and the predicted JuA targets. (G) Proin–protein interaction (PPI) analysis of the overlapping genes, interleukin (*IL*)-*6*, ATPase Na^+^/K^+^ transporting subunit alpha 2 (*ATP1A2*), and hydroxysteroid 11-beta dehydrogenase 1 (*HSD11B1*). (H) Prognostic analyses of IL-6, ATP1A2, and HSD11B1 in The Cancer Genome Atlas (TCGA) bladder cancer cohort.

The GSE133624 dataset was downloaded from the Gene Expression Omnibus database. Principal component analysis results of the GSE133624 dataset are shown in Figure 1A, where red circles indicate the cases (cancer tissues), and cyan circles indicate the controls (Ctrls; normal tissues). Although most areas showed no overlap, we observed a partially overlapping area between the case and control groups, confirming specific differences in the transcriptomes of cancer and normal tissues. Volcano plot shows the DEGs. In total, 1845 DEGs were identified, 451 of which were upregulated and 1394 were downregulated (Figure 1B). The heat map shows the top 10 most significantly upregulated and downregulated DEGs (Figure 1C). Pathway enrichment analysis showed that the DEGs were enriched mainly in the phosphoinositide 3-kinase/protein kinase B signaling, cytokine–cytokine receptor, and cell adhesion molecule pathways (Figure 1D and E).

To explore potential JuA drug targets among the DEGs detected in bladder cancer, we retrieved JuA targets from the Encyclopedia of Traditional Chinese Medicine database and examined their overlap with the detected DEGs. Three potential targets (IL6, ATP1A2, and HSD11B1) were identified (Figure 1F). Using the GENEMANIA database, we constructed a protein–protein interaction (PPI) network of the overlapping genes. Notably, the three potential target genes were associated with many other genes, such as *IL6R*, *ATP1B2*, and *ATP1B1* (Figure 1G).

To evaluate the clinical prognostic value of the potential target genes (*IL6*, *ATP1A2*, and *HSD11B1*), Kaplan–Meier survival curves were constructed based on The Cancer Genome Atlas (TCGA) database for OS analysis. No significant correlation was observed between IL-6 levels and OS. However, the groups with high ATP1A2 and HSD11B1 levels exhibited significantly low OS (Figure 1H), suggesting that ATP1A2 and HSD11B1 as potential biomarkers of poor prognosis. HSD11B1 acts as a tumor suppressor in renal^18^ and pancreatic^19^ cancers. Interestingly, high HSD11B1 expression was associated with poor prognosis in this study. This may be due to the different responses of the body to anticancer drugs, increasing HSD11B1 levels to exert anticancer effects. Therefore, HSD11B1 was not selected for further analysis. ATP1A2, which is highly expressed in many cancer types,^20-23^ shows potential as a therapeutic target. Moreover, no study has reported its specific role in bladder cancer to date. Therefore, we focused on ATP1A2 in subsequent experiments.

### ATP1A2 overexpression promotes mitochondrial energy metabolism and inhibits apoptosis in the 5637 cells

**Figure 2.**
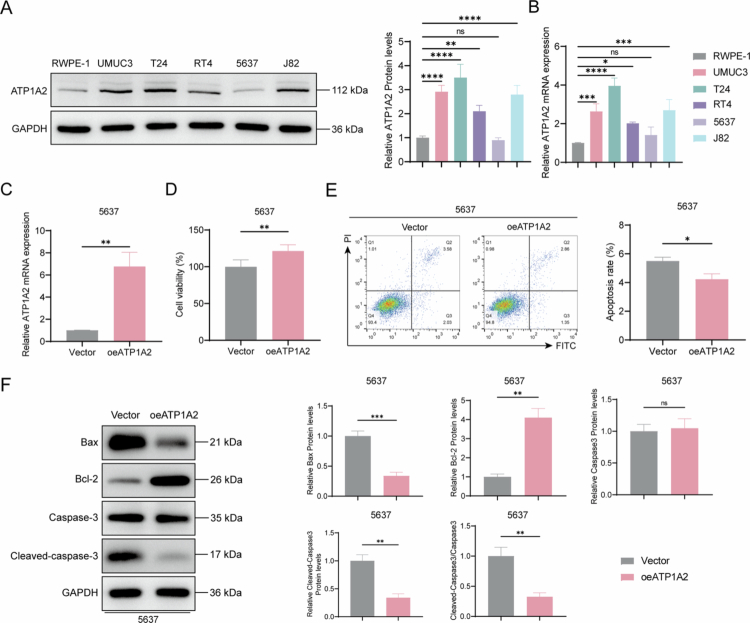
ATP1A2 overexpression promotes the proliferation and inhibits the apoptosis of bladder cancer cells. (A) The relative expression levels of ATP1A2 protein in RWPE-1 normal human prostate epithelial cells and different bladder cancer cell lines (UMUC3, 5637, RT4, T24, and J82) were detected by Western blotting (*n* = 6). (B) Reverse transcription quantitative polymerase chain reaction (RT-qPCR) was used to detect the relative expression levels of mRNA in RWPE-1 normal human prostate epithelial cells and different bladder cancer cell lines (UMUC3, 5637, RT4, T24 and J82) (*n* = 6). (C) ATP1A2 overexpression plasmid was transfected into the 5637 cells for 48  h, and the relative *ATP1A2* mRNA levels were determined via RT-qPCR (*n* = 6). (D) Cell counting kit (CCK)-8 assay was used to assess the 5637 cell viability 48  h after ATP1A2 overexpression plasmid vector transfection (*n* = 6). (E) Flow cytometry with fluorescein isothiocyanate (FITC)/propidium iodide (PI) double staining was performed to assess the 5637 cell apoptosis ratio 48  h after ATP1A2 overexpression plasmid vector transfection (*n* = 6). (F) Western blotting analysis was used to determine the relative expression levels of the apoptosis-related proteins, Bcl2-associated X (Bax), Bcl-2, caspase-3, and cleaved-caspase-3 (*n* = 6). *P < 0.05, **P < 0.01, ***P < 0.001, ****P < 0.0001; ns, *P* > 0.05.

**Figure 3.**
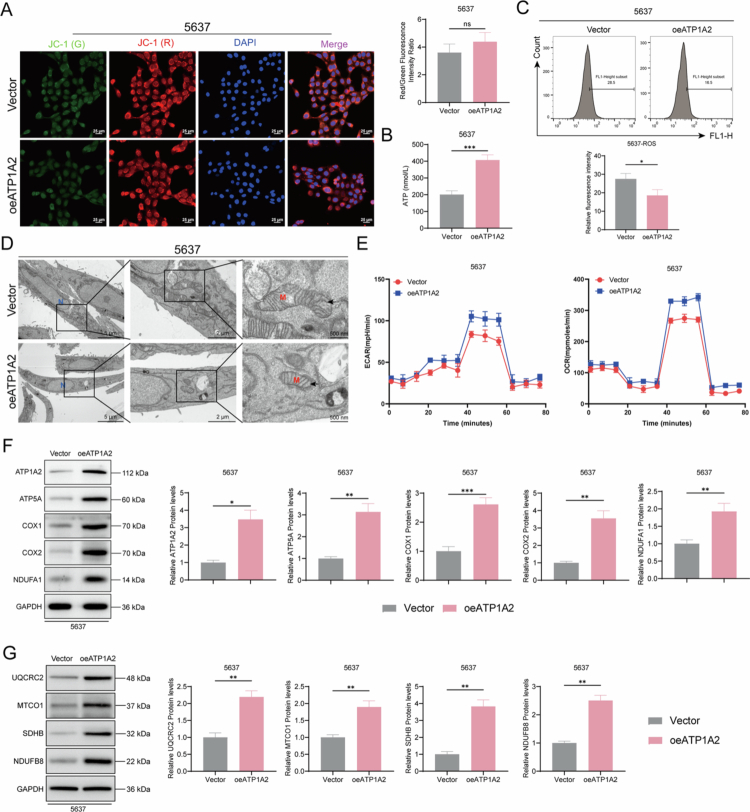
ATP1A2 overexpression enhances energy metabolism in bladder cancer cells. (A) The 5,5′,6,6′-tetrachloro-1,1′,3,3′-tetraethylbenzimidazolcarbocyanine iodide (JC-1) mitochondrial membrane potential fluorescence probe assay was used to detect the mitochondrial membrane potential in 5637 cells 48  h after ATP1A2 overexpression plasmid vector transfection (×400; scale bar: 25 μm) (*n* = 6). (B) Changes in ATP levels in 5637 cells after transfection (*n* = 6). (C) Changes in intracellular reactive oxygen species (ROS) levels in 5637 cells after transfection were determined via flow cytometry (*n* = 6). (D) Morphological changes in the mitochondrial ultrastructure of 5637 cells after transfection were observed via transmission electron microscopy (black arrows, normal mitochondria; red arrows, damaged mitochondria; N, nucleus; M, mitochondria; ×3000, scale bar: 5 μm; ×8000, scale bar: 2 μm; ×20,000, scale bar: 500 nm) (*n* = 6). (E) Measurement of OCR and ECAR in 5637 cells following ATP1A2 overexpression (*n* = 6). (F) Western blotting was used to determine the changes in the expression levels of mitochondrial energy metabolism-related proteins (ATP1A2, ATP5A, COX1, COX2, and NADH:ubiquinone oxidoreductase subunit A1 [NDUFA1]) (*n* = 6). (G) Western blotting was used to determine the changes in the expression levels of mitochondrial energy metabolism-related proteins (UQCRC2, MTCO1, SDHB, and NDUFB8) (*n* = 6). *P < 0.05, **P < 0.01, ***P < 0.001, and ****P < 0.0001; ns, *P* > 0.05.

We assessed ATP1A2 expression levels using different bladder cancer cell lines. Compared to those in RWPE-1 normal human prostate epithelial cells, ATP1A2 levels were significantly upregulated in the UMUC3, 5637, RT4, and J83 bladder cancer cell lines, with the most obvious upregulation observed in the T24 cells. In contrast, ATP1A2 levels were significantly downregulated in the 5637 cells (Figure 2A and 2B). To determine the impacts of differential ATP1A2 expression on bladder cancer cells, we established an ATP1A2 overexpression cell line from 5637 cells with low ATP1A2 expression. RT-qPCR revealed significantly increased *ATP1A2* mRNA levels in the oeATP1A2 group (transfected with the ATP1A2 overexpression plasmid), confirming the successful establishment of a cell line with high ATP1A2 expression (Figure 2C). The CCK8 assay results indicated that ATP1A2 overexpression significantly enhanced the cell viability (Figure 2D) and decreased the cell apoptosis rate (Figure 2E). Western blotting analysis of apoptosis-related protein levels revealed that ATP1A2 overexpression significantly decreased the Bcl2-associated X (Bax) levels and cleaved-caspase to 3/caspase-3 ratio and significantly increased the Bcl-2 levels (Figure 2F), suggesting that ATP1A2 overexpression inhibits 5637 cell apoptosis. Functional experiments were performed to explore the relationship between ATP1A2 and mitochondrial energy metabolism. The 5,5′,6,6′-tetrachloro-1,1′,3,3′-tetraethylbenzimidazolcarbocyanine iodide fluorescence probe assay showed that ATP1A2 overexpression significantly increased the mitochondrial membrane potential (Figure 3A) and intracellular ATP levels (Figure 3B) and significantly decreased the ROS levels (Figure 3C) in 5637 cells. Transmission electron microscopy (TEM) observation of the cell ultrastructure showed that, under ATP1A2 overexpression conditions, the mitochondria exhibited an intact morphology, with cristae arranged in an orderly manner, which was consistent with the morphological characteristics of the control group (Figure 3D). We also observed the changes in the intracellular levels of OCR and ECAR. The results showed that, compared with the vector group, the levels of OCR and ECAR increased significantly after the over-expression of ATP1A2. These findings suggest that the overall cellular energy metabolism was enhanced. (Figure 3E). To further confirm the relationship with mitochondrial energy metabolism, we determined the expression levels of mitochondrial energy metabolism-related proteins using western blotting. ATP1A2 overexpression significantly increased the relative protein levels of ATP1A2, ATP5A, COX1, COX2, NADH:ubiquinone oxidoreductase subunit A1, UQCRC2, MTCO1, SDHB, and NDUFB8 (NDUFA1; Figure 3F and 3G). These results confirmed that ATP1A2 overexpression enhanced mitochondrial energy metabolism in the 5637 cells.

### ATP1A2 knockdown inhibits mitochondrial energy metabolism and induces apoptosis in T24 cells

**Figure 4.**
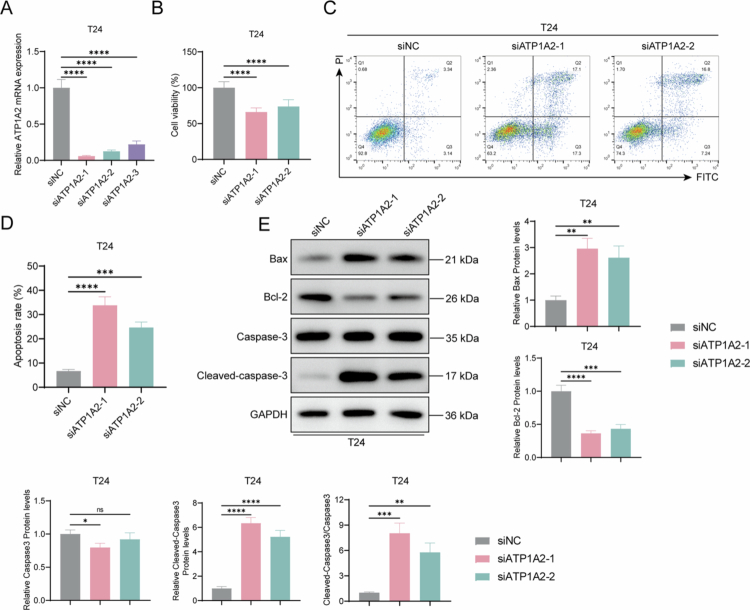
ATP1A2 knockdown inhibits the proliferation and induces the apoptosis of bladder cancer cells. (A) After transfecting interfering RNAs (siATP1A2-1, siATP1A2-2, and siATP1A2-3) into the T24 cells for 48  h, the relative *ATP1A2* mRNA levels were determined via RT-qPCR (*n* = 6). (B) After siRNA transfection into the T24 cells for 48  h, changes in cell viability were assessed using the CCK8 assay (*n* = 6). (C) After siRNA transfection for 48 h, changes in the apoptosis ratio of T24 cells were analyzed via flow cytometry with FITC/PI double staining (*n* = 6). (D) Histogram showing the statistical analysis of the apoptosis rate (*n* = 6). (E) Western blotting analysis was used to determine the relative expression levels of the apoptosis-related proteins, Bax, Bcl-2, caspase-3, and cleaved-caspase-3 (*n* = 6). *P < 0.05, **P < 0.01, ***P < 0.001, and ****P < 0.0001; ns, *P* > 0.05.

**Figure 5.**
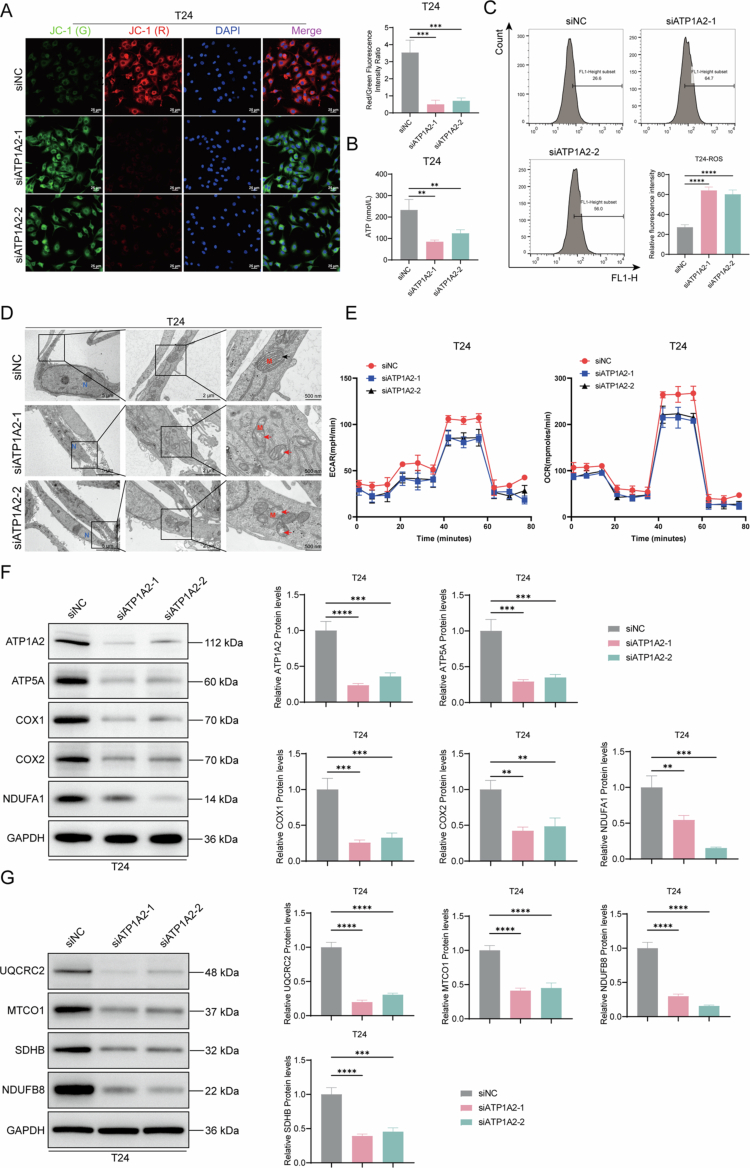
ATP1A2 knockdown inhibits energy metabolism in bladder cancer cells. (A) JC-1 mitochondrial membrane potential fluorescence probe assay was used to observe the changes in the intracellular mitochondrial membrane potential of T24 cells 48  h after siRNA transfection (×400; scale bar: 25 μm) (*n* = 6). (B) Changes in ATP levels in T24 cells were assessed after transfection (*n* = 6). (C) Changes in intracellular ROS levels in T24 cells after transfection were analyzed via flow cytometry (*n* = 6). (D) Morphological changes in the mitochondrial ultrastructure of T24 cells after transfection were observed via transmission electron microscopy (black arrows, normal mitochondria; red arrows, damaged mitochondria; N, nucleus; M, mitochondria; ×3000, scale bar: 5 μm; ×8000, scale bar: 2 μm; ×20,000, scale bar: 500 nm) (*n* = 6). (E) Measurement of the OCR and ECAR in 5637 cells after ATP1A2 knockdown (*n* = 6). (F) Western blotting was performed to determine the expression levels of mitochondrial energy metabolism-related proteins (ATP1A2, ATP5A, COX1, COX2, and NDUFA1) (*n* = 6). (G) Western blotting was used to determine the changes in the expression levels of mitochondrial energy metabolism-related proteins (UQCRC2, MTCO1, SDHB, and NDUFB8) (*n* = 6). *P < 0.05, **P < 0.01, ***P < 0.001, and ****P < 0.0001; ns, *P* > 0.05.

To confirm that ATP1A2 expression levels affect bladder cancer cells, we established an ATP1A2 knockdown cell line from T24 cells. RT-qPCR revealed that siATP1A2-1, siATP1A2-2, and siATP1A2-3 significantly reduced the relative *ATP1A2* mRNA levels, with siATP1A2-1 and siATP1A2-2 causing the most notable reduction. Therefore, the interference sequences siATP1A2-1 and siATP1A2-2 were used for subsequent experiments using ATP1A2 knockdown cell models (Figure 4A). ATP1A2 knockdown significantly decreased the cell viability (Figure 4B) and increased the cell apoptosis ratio (Figure 4C and D). Western blotting analysis revealed significantly increased Bax protein levels and cleaved-caspase-3/caspase-3 ratio and significantly decreased Bcl-2 protein levels after ATP1A2 knockdown in T24 cells (Figure 4E), suggesting that ATP1A2 knockdown induces apoptosis in T24 cells. ATP1A2 knockdown in T24 cells significantly decreased the mitochondrial membrane potential (Figure 5A) and intracellular ATP levels (Figure 5B) and increased the ROS levels (Figure 5C). TEM observation further revealed that ATP1A2 knockdown caused characteristic morphological changes in the mitochondria, including damage to the mitochondrial membrane structure and disordered cristae arrangement, in T24 cells. These morphological features are consistent with previously reported mitochondrial changes during ferroptosis in cells^24^ (Figure 5D). We also observed the changes in the intracellular OCR and ECAR levels. The results showed that, compared with the siNC group, the levels of OCR and ECAR decreased significantly after ATP1A2 knockdown. These findings indicate that the overall energy metabolism of the cells was inhibited (Figure 5E). Western blotting was performed to detect the proteins involved in mitochondrial energy metabolism. ATP1A2 knockdown significantly decreased the relative levels of ATP1A2, ATP5A, COX1, COX2, NDUFA1, UQCRC2, MTCO1, SDHB, and NDUFB8 (Figure 5F and 5G). These results suggest that ATP1A2 knockdown inhibits mitochondrial energy metabolism in T24 cells.

### JuA inhibits ATP1A2-induced T24 cell apoptosis

**Figure 6.**
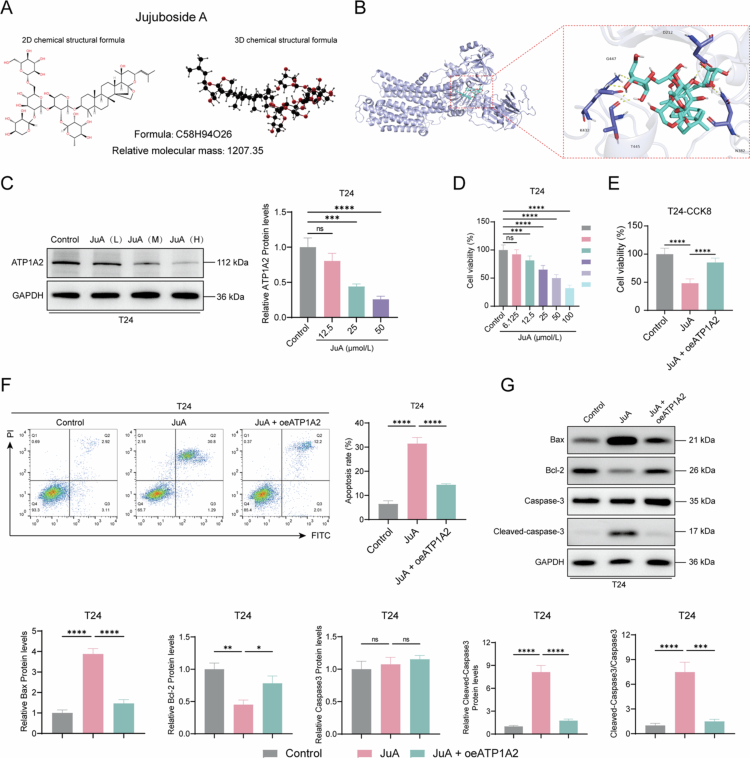
JuA inhibits ATP1A2-induced bladder cancer cell apoptosis. (A) Two-dimensional (2D) and three-dimensional (3D) chemical structures of JuA and their relative molecular masses. (B) Molecular docking was performed between JuA and the ATP1A2 protein (as shown in the figure, the protein was represented in blue cartoon, and the compound was shown in cyan sticks). (C) T24 cells were treated with different concentrations of JuA (12.5, 25, and 50 μmol/L) for 48  h. Changes in relative ATP1A2 protein levels were subsequently assessed via western blotting (*n* = 6). (D) After treating T24 cells with different concentrations of JuA (6.125, 12.5, 25, 50, and 100 μmol/L) for 48  h, changes in cell viability were analyzed via CCK8 assay (*n* = 6). (E) After treating with 50 μmol/L of JuA for 48  h, transfecting with the ATP1A2 overexpression plasmid, and culturing for another 48  h, changes in T24 cell viability were analyzed via CCK8 assay (*n* = 6). (F) After treatment with JuA (50 μmol/L) and overexpression plasmid transfection, changes in the cell apoptosis ratio were assessed via flow cytometry with FITC/PI double staining (*n* = 6). (G) After treatment with JuA (50 μmol/L) and overexpression plasmid transfection, the relative expression levels of the apoptosis-related proteins, Bax, Bcl-2, caspase-3, and cleaved-caspase-3, were determined via western blotting (*n* = 6). *P < 0.05, **P < 0.01, ***P < 0.001, and ****P < 0.0001; ns, *P* > 0.05.

We successfully demonstrated that ATP1A2 knockdown induced apoptosis and inhibited mitochondrial energy metabolism in cells. The two- and three-dimensional molecular structures of JuA are shown in Figure 6A. Molecular docking results indicate that the compound forms a stable interaction network with the target protein, involving a total of 6 hydrogen bonds with key residues, including D212, N382, K432, T445, and G447. These hydrogen bonds likely involve both main-chain and side-chain atoms, suggesting that the compound effectively occupies the binding pocket of the protein and engages in specific interactions with critical site residues. Furthermore, the docking score for this binding is 9.1  kcal/mol, which reflects a high theoretical binding affinity between the compound and the protein (Figure 6B). To confirm the potential targeting relationship between JuA and ATP1A2, we treated T24 cells with different concentrations of JuA (6.125, 12.5, 25, 50, and 100 μmol/L). T24 cell viability gradually decreased with increasing JuA concentration in a concentration-dependent manner. Different concentrations of JuA (25, 50, and 100 μmol/L) also gradually decreased the ATP1A2 levels (Figure 6C and 6D). To verify that JuA exerts anticancer effects by targeting and inhibiting ATP1A2, we transfected an ATP1A2 overexpression plasmid into T24 cells after JuA treatment. CCK8 assay and flow cytometry showed that JuA significantly decreased the viability and induced the apoptosis of T24 cells, whereas transfection with the ATP1A2 overexpression plasmid reversed these effects. Furthermore, western blotting revealed that JuA significantly upregulated the Bax protein levels and cleaved-caspase-3/caspase-3 ratio and significantly downregulated the Bcl-2 protein levels in T24 cells (Figure 6E–G). These results confirmed that JuA targeted and inhibited ATP1A2 to induce T24 cell apoptosis.

### JuA inhibits ATP1A2 expression and mitochondrial energy metabolism in T24 cells

**Figure 7.**
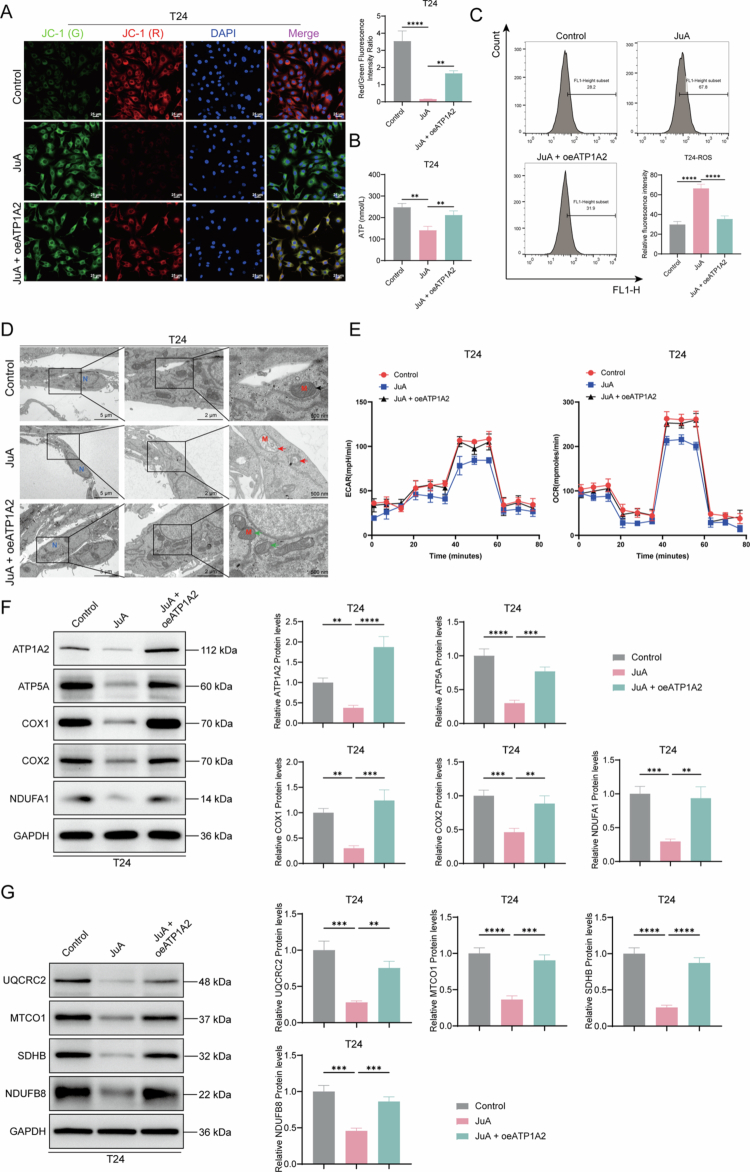
JuA inhibits ATP1A2-mediated mitochondrial energy metabolism disruption in bladder cancer cells. (A) After treating with 50 μmol/L of JuA for 48  h, transfecting with the ATP1A2 overexpression plasmid, and culturing for another 48  h, changes in the mitochondrial membrane potential of T24 cells were assessed via a JC-1 mitochondrial membrane potential fluorescence probe assay (*n* = 6). (B) After treatment with JuA (50 μmol/L) and overexpression plasmid transfection, changes in the intracellular ATP levels were determined via an ATP assay (*n* = 6). (C) After treatment with JuA (50 μmol/L) and overexpression plasmid transfection, changes in the intracellular ROS levels in T24 cells were assessed via flow cytometry (*n* = 6). (D) After treatment with JuA (50 μmol/L) and overexpression plasmid transfection, morphological changes in the mitochondrial ultrastructure of T24 cells were observed via transmission electron microscopy (black arrows, normal mitochondria; red arrows, damaged mitochondria; *N*, nucleus; M, mitochondria; × 3000, scale bar: 5 μm; × 8000, scale bar: 2 μm; × 20,000, scale bar: 500 nm) (*n* = 6). (E) After treatment with JuA, the ATP1A2 overexpression plasmid was transfected. At the end of the treatment, the OCR and ECAR in each group of T24 cells were measured (*n* = 6). (F) After treatment with JuA (50 μmol/L) and overexpression plasmid transfection, changes in the expression of mitochondrial energy metabolism-related proteins (ATP1A2, ATP5A, COX1, COX2, and NDUFA1) were determined via western blotting (*n* = 6). (G) Western blotting was used to determine the changes in the expression levels of mitochondrial energy metabolism-related proteins (UQCRC2, MTCO1, SDHB, and NDUFB8) (*n* = 6). *P < 0.05, **P < 0.01, ***P < 0.001, and ****P < 0.0001; ns, *P* > 0.05.

Analysis of mitochondrial energy metabolism revealed that JuA significantly decreased the mitochondrial membrane potential and intracellular ATP content but increased the ROS levels in T24 cells. However, ATP1A2 overexpression reversed these pharmacological effects of JuA (Figure 7A–C). TEM showed that JuA treatment caused characteristic morphological changes in the mitochondria, including damage to the mitochondrial membrane structure and disordered cristae arrangement, in T24 cells (Figure 7D). The detection results of OCR and ECAR showed that JuA could significantly inhibit OCR and ECAR, thus suppressing cellular energy metabolism. However, the inhibitory effect of JuA was counteracted after the overexpression of ATP1A2 (Figure 7E). However, ATP1A2 overexpression restored mitochondrial morphology. Subsequently, western blotting was performed to determine the mitochondrial energy metabolism-related protein levels. JuA significantly decreased the relative expression levels of ATP1A2, ATP5A, COX1, COX2, NDUFA1, UQCRC2, MTCO1, SDHB, and NDUFB8 (Figure 7F and 7G). However, ATP1A2 overexpression reversed this effect. These findings confirmed that JuA inhibited ATP1A2 expression, thereby suppressing mitochondrial energy metabolism in T24 cells.

## Discussion

Effective treatment of bladder cancer is limited by several challenges. Traditional chemotherapy is highly toxic, increasing the patient drug resistance. Although novel therapeutics, such as immune checkpoint inhibitors and antibody–drug conjugates, are effective, they exhibit various limitations, such as strong target dependence, adverse reactions, and high treatment costs. Owing to their potential for multi-target regulation and low toxicity, natural products have been explored for new antitumor drugs.^4^^,^^5^ In this study, in vitro experiments confirmed the inhibitory effects of JuA on bladder cancer cells and preliminarily indicated that JuA induces apoptosis by regulating ATP1A2 expression, which affects mitochondrial energy metabolism. Bcl-2 and Bax are key regulatory factors determining the initiation of mitochondrial apoptosis via competition. Upon initiation (when Bax becomes dominant), the downstream caspase cascade is activated, ultimately leading to the cleavage and activation of caspase-3 into cleaved-caspase-3, which executes the cell disassembly program. These four proteins are the core targets in apoptosis research. Their expression levels, activation status (especially cleaved-caspase-3), and ratios (Bax/Bcl-2) directly reflect the apoptotic status and regulatory mechanisms of cells.^25^ In this study, ATP1A2 overexpression reduced the Bax and cleaved-caspase-3 levels and increased the Bcl-2 levels, thereby inhibiting the apoptosis of 5637 cells. In terms of signaling pathway mechanisms, ATP1A2 can inhibit the TGFβ/SMAD signaling pathway.^14^ However, in the context of cancer-related scenarios, whether ATP1A2 is involved in regulating classical pathways such as the PI3K/AKT and MAPK pathways has not yet been reported. Notably, in other disease models, the transcriptional regulation of ATP1A2 has been proven to rely on the activation of the PI3K/AKT and NFκB pathways.^26^ This finding further suggests that the mechanism of the role of ATP1A2 in signal pathways in cancer remains unclear, which is of great research value and potential. This will also be one of the key directions for our future exploration.

Mitochondrial energy metabolism disorders result from the combined effects of multiple factors. Excessive ROS generated by oxidative stress directly damage the mitochondria or activate specific pathways, significantly decreasing the mitochondrial membrane potential (ΔΨm). This disrupts the proton gradient essential for ATP synthesis. Observation of the mitochondrial ultrastructure using electron microscopy revealed various pathological morphologies, such as swelling, cristae breakage, and disappearance. Such structural damage not only directly impairs the spatial conformations and functions of electron transport chain complexes but also exacerbates the collapse of ΔΨm, severely disrupting mitochondrial energy metabolism, specifically ATP generation via oxidative phosphorylation, thereby impairing the cellular energy supply.^27^ Here, ATP1A2 knockdown significantly increased the oxidative stress, decreased the ΔΨm, and disrupted the mitochondrial ultrastructure in bladder cancer cells. Collectively, these findings provide direct experimental evidence for the regulation of mitochondrial energy metabolism by ATP1A2. In tumor cells, alterations in mitochondrial energy metabolism are often associated with several characteristics, such as cell proliferation, apoptosis, and drug resistance. ATP1A2 plays crucial roles in maintaining the cell membrane ion gradient and signal transduction.^11-13^ The mitochondrial oxidative phosphorylation system is a highly coordinated electron transport chain. Its core consists of four protein complexes (I, II, III, IV) and ATP synthase (Complex V), which work together to establish a proton gradient and synthesize ATP. Complex I (NADH:ubiquinone oxidoreductase) is composed of subunits such as NDUFA1 and NDUFB8. It catalyzes the transfer of electrons from NADH to ubiquinone while pumping out protons. Through its iron-sulfur protein subunit SDHB, Complex II (succinate dehydrogenase) subsequently transfers electrons from succinate to ubiquinone without pumping protons across the membrane. The electrons of reduced ubiquinone are transferred to cytochrome c via Complex III (ubiquinone – cytochrome c reductase, with the core subunit UQCRC2), further strengthening the proton gradient in this process. Complex IV (cytochrome c oxidase), through its catalytic center MTCO1 (i.e., COX1) and structural subunit COX2, finally transfers electrons to oxygen and completes the last proton transfer. The transmembrane proton‒motive force thus established is ultimately utilized by ATP synthase (Complex V, with its catalytic head being ATP5A) to drive the combination of ADP and inorganic phosphate to generate ATP.^28^^,^^29^ In this study, ATP1A2 overexpression enhanced the relative protein levels of ATP1A2, ATP5A, COX1, COX2, NDUFA1, UQCRC2, MTCO1, SDHB, and NDUFB8, promoting mitochondrial energy metabolism. Conversely, ATP1A2 knockdown inhibited mitochondrial energy metabolism. To the best of our knowledge, to date, no study has reported the regulatory relationship between ATP1A2 and mitochondrial energy metabolism in bladder cancer. Notably, this study revealed that ATP1A2 influenced mitochondrial energy metabolism in bladder cancer cells, suggesting a new avenue for controlling the growth and promoting the apoptosis of tumor cells.

JuA exerts inhibitory effects on bladder cancer cells; its unique action mechanism of influencing mitochondrial energy metabolism by targeting ATP1A2 strongly supports its therapeutic application for cancer. However, this study has several limitations. First, this study primarily involved in vitro cell experiments. Although the action mechanisms of JuA were preliminarily examined, its efficacy, pharmacokinetics, and safety could not be assessed because of the lack of in vivo models. In-vivo experiments will be the focus of our subsequent research, which can provide strong support for our conclusions. Second, although ATP1A2 was identified as a potential target of JuA via database analysis and preliminary experiments, its direct interactions and detailed regulatory mechanisms remain ambiguous, warranting further in-depth research. Specifically, the roles of specific signaling pathways and PPIs need to be further investigated. As tumor onset and progression are complex processes involving abnormalities in multiple genes and signaling pathways, targeting ATP1A2 alone is possibly insufficient for effective bladder cancer treatment. Therefore, future studies should assess the combined applications of JuA and other agents and explore additional targets and synergistic mechanisms to improve the treatment efficacy and overcome drug resistance in bladder cancer.

## Conclusion

In this study, we used an online database to identify potential JuA targets for bladder cancer treatment. In in vitro experiments, we explored and verified the significant inhibitory effects of JuA on bladder cancer cells. Notably, JuA effectively regulated ATP1A2 expression, thereby influencing mitochondrial energy metabolism and triggering apoptosis in bladder cancer cells. This innovative discovery provides a solid experimental foundation for the development of safe and effective ATP1A2-targeted therapeutic strategies, facilitating the precise and personalized treatment of bladder cancer in the future.

## Materials and methods

### Data grouping

The Gene Expression Omnibus database (https://www.ncbi.nlm.nih.gov/geo/) was used to obtain expression data. The GSE133624 dataset (transcriptomic analysis of gene expression levels in human urothelial carcinoma of the bladder and adjacent normal tissues) was used to explore the differential expression between the bladder cancer disease (case; bladder cancer tissues) and control (Ctrl; adjacent normal tissues) groups. In the GSE133624 dataset, the case-to-control sample ratio was 36:29.

### Differential mRNA expression analysis using the GSE133624 dataset

Differential expression between the case and control groups was analyzed using the “limma” package.^30^ Screening was performed according to the following criteria: |log FC| > 1 and *P* < 0.05. In total, 1845 DEGs, including 451 upregulated and 1394 downregulated DEGs, were identified. The R packages “FactoMineR” and “factoextra” were used to conduct principal component analysis of the two groups based on the detected DEGs.^31^ The R package “pheatmap” was used to draw a cluster heatmap of DEGs,^32^ and the R package “ggplot2” was used to visualize the log2FC values and DEG significance.^33^

### Gene ontology (GO) and kyoto encyclopedia of genes and genomes (KEGG) functional enrichment analyses of DEGs in the GSE133624 dataset

The R package “clusterProfiler” was used for GO and KEGG functional enrichment analyses of the DEGs obtained from the GSE133624 dataset to determine the associated GO functions and KEGG pathways and note the top-ranked results.^34^

### Screening of key target genes of JuA in bladder cancer

“Jujuboside A” was used as the keyword to search for JuA targets in the Encyclopedia of Traditional Chinese Medicine database (http://www.tcmip.cn/ETCM/index.php/Home/Index/index.html). The JuA targets and identified DEGs were overlapped. The R package “Venn Diagram” was used to draw a Venn diagram.^35^ The GENEMANIA database (http://genemania.org/) was used to construct a PPI network of the overlapping genes. Using survival data from the TCGA-Bladder Urothelial Carcinoma cohort, Kaplan–Meier analysis was performed to evaluate the correlation between the target gene (*IL-6*, *ATP1A2*, and *HSD11B1*) levels and patient OS. The significance threshold was set at *P* < 0.05.^36^

### Molecular docking

The structures of the active compounds were downloaded from PubChem and imported into ChemBio3D 14.0 software to adjust the spatial conformation of the active ingredients, calculate the optimization of energy. The three-dimensional crystal structure of the target protein was downloaded from the UniProt. The water molecules and organic matter in the target protein were removed by PyMOL, and then the target protein was imported into AutoDockTools1.5.6 for hydrogenation, charge distribution, and atomic type addition. AutoDock Vina was used for molecular docking, and the docking results were plotted with Discovery Studio 2021 and PyMOL 2.6.1.

### Cell culture

The SV-HUC-1 normal human bladder epithelial cell line (CL-0222) and UMUC3 (CL-0463), 5637 (CL-0002), RT4 (CL-0431), T24 (CL-0227), and J82 (CL-0125) human bladder cancer cell lines were purchased from Wuhan Pricella Biotechnology Co., Ltd. All the cell lines were purchased from Wuhan Pricella Biotechnology Co., Ltd. (Wuhan, China) and were supplied with authentication via short tandem repeat (STR) profiling and confirmed to be free of mycoplasma contamination. All the cells were cultured in high-glucose Dulbecco’s modified Eagle’s medium (DMEM; PM150210B; Wuhan Pricella Biotechnology Co., Ltd.) supplemented with 10% fetal bovine serum and 1% penicillin–streptomycin. The cells were placed in a cell incubator (120300; Thermo Fisher Scientific, China) at 37 °C with 5% CO₂. The medium was replaced every other day. After being cultured until the exponential growth phase, the cells were used for subsequent experiments.

### Cell transfection and grouping

Next, the 5637 and T24 cells in the exponential growth phase were collected. Trypsin (2 mL) was added, and the cells were incubated for 3 min. Next, 3 mL of high-glucose DMEM was added, and the cell suspension was collected in a centrifuge tube and centrifuged at 1200 × *g* for 3 min. After adding 3 mL of high-glucose DMEM, the cell density was calculated using a cell counter (A49866; Thermo Fisher Scientific). The cells were seeded in a 6-well plate at a density of 1 × 10⁵ cells/well and incubated overnight. Specific interfering RNAs and overexpression plasmids were designed for *ATP1A2*. The next day, according to the transfection reagent manufacturer’s instructions (11668500; Thermo Fisher Scientific), a mixture of the overexpression plasmid and transfection reagent was added to the 5637 cells, whereas a mixture of interfering RNA and transfection reagent was added to the T24 cells. The cells were incubated for 4 h. Then, the medium was replaced, and the cells were further incubated for 48 h. RT-qPCR and western blotting analyses were performed to confirm the establishment of the ATP1A2 overexpression and knockdown cell models.

All siRNA interference sequences and overexpression plasmids are listed in Table S1.

### Cell grouping to verify the effects of high and low ATP1A2 levels on bladder cancer progression

The 5637 cells were grouped into two sets: overexpression control (denoted as vector) and ATP1A2 overexpression (designated as oe-ATP1A2) groups. T24 cells were divided into three groups: knockdown control (siNC), first ATP1A2 knockdown (termed siATP1A2-1), and second ATP1A2 knockdown (siATP1A2-2) groups. To validate the pharmacological effects of JuA (HY-N0659; MedChemExpress, China), bladder cancer cells were treated with different concentrations of JuA and divided into four groups: control, JuA-L (12.5 μmol/L), JuA-M (25 μmol/L), and JuA-H (50 μmol/L) groups. To determine whether the pharmacological effects of JuA are reliant on ATP1A2, the cells were organized into three groups: control, JuA-H (50 μmol/L), and JuA-H (50 μmol/L) plus oe-ATP1A2 groups.

### CCK8 assay

Next, 5637 and T24 cells in the exponential growth phase were seeded in a 96-well plate at a cell density of 3000 cells/well and incubated overnight. After treating the cells according to different grouping requirements, 10  μL of CCK8 solution was added to each well, and the plates were incubated for an additional 3 h. Then, the plates were placed in a microplate reader (VLBLATGD2; Thermo Fisher Scientific), and the optical density was measured at 450 nm. The data were used for statistical analyses.

### Flow cytometry for ROS detection

T24 and 5637 cells were collected during the exponential growth phase. After treatment according to their groupings, the cells were washed 2–3 times with pre-cooled phosphate-buffered saline (PBS). According to the instructions of the ROS detection kit (S0033S; Beyotime Biotech Inc., China), 2′,7′-dichlorofluorescin diacetate probe was added to the cell suspension at a final concentration of 4 μmol/L. Then, the cells were incubated in an incubator at 37 °C with 5% CO₂ for 30 min. After incubation, the fluorescence intensity of intracellular ROS was detected using a flow cytometer (A24858; Thermo Fisher Scientific). The average fluorescence intensity of the ROS in each group was analyzed and statistically calculated using the ImageJ 1.5.2a software.

### Flow cytometry for cell apoptosis detection

After treating the exponential growth phase cells according to their grouping, the 5637 and T24 cells were collected after trypsin digestion. The cell concentration was adjusted to approximately 1 × 10⁶ cells/mL. Then, 100  μL of the cell suspension was added to the flow cytometry tube. According to the operating procedures of the annexin V-fluorescein isothiocyanate/propidium iodide apoptosis detection kit (C1062L; Beyotime Biotech Inc.), 5  μL of annexin V-fluorescein isothiocyanate and 5  μL of propidium iodide dye were successively added, followed by incubation in the dark for 15  min. Changes in apoptosis were detected via flow cytometry. The data were saved, and statistical analyses were conducted using the FlowJo v10.8.1 software.

### Electron microscopy for mitochondrial structure observation

The 5637 and T24 cells in the exponential growth phase were treated according to their grouping. The cells were fixed with 2.5% glutaraldehyde for 4 h, washed three times with PBS, and dehydrated using graded ethanol (30, 50, 70, 80, 90, 95, and 100%; 10 min each). After acetone replacement, the cells were embedded in epoxy resin. The embedded block was polymerized and solidified, and ultrathin sections of approximately 70-nm thickness were cut using an ultramicrotome. The sections were placed on copper grids and double-stained with uranyl acetate and lead citrate for 15 min each. Finally, the sections were observed and photographed using a transmission electron microscope (Talos F200X G2; Thermo Fisher Scientific).

### ATP content determination

After treatment according to their grouping, 5637 and T24 cells in the exponential growth phase were centrifuged, and their supernatants were used for ATP content determination. According to the operation instructions of the ATP fluorescence quantitative detection kit (CB11233-Hu; COIBO BIO, China), the supernatants were mixed with the ATP detection reagent in a specific reaction system and incubated for 1 h. Then, the optical density was measured at 450 nm using a microplate reader for subsequent analysis.

### Mitochondrial membrane potential detection

After the 5637 and T24 cells in the exponential growth phase were subjected to group-based treatments, according to the requirements of the kit (C2003S; Beyotime Biotech Inc.), 5,5′,6,6′-tetrachloro-1,1′,3,3′-tetraethylbenzimidazolcarbocyanine iodide staining working solution was added, and the cells were incubated in a cell incubator at 37 °C with 5% CO₂ for 30 min. Then, the cells were washed 2–3 times with PBS and observed and photographed using a fluorescence microscope (AMF5000; Thermo Fisher Scientific). Changes in fluorescence intensity were analyzed using the ImageJ 1.5.2a software.

### RT-qPCR

Total RNA was extracted from treated 5637 and T24 cells in the exponential growth phase using the TRIzol reagent (R1100; Beijing Solarbio Science & Technology Co., Ltd., China), according to the kit instructions. The RNA concentration and purity were determined using a spectrophotometer (840-317500; Thermo Fisher Scientific). Reverse transcription was performed using a reverse transcription kit (RR037B; TaKaRa Biotechnology Co., Ltd., China). Specific primers were designed based on the mRNA sequences of the target and reference genes. For RT-PCR using cDNA as the template, the reaction system was prepared according to the instructions of the SYBR Green fluorescence quantitative PCR kit (RR066B; TaKaRa Biotechnology Co., Ltd.). The reaction conditions were as follows: 42 °C for 5  min, 95 °C for 10 s, followed by 40 cycles of 95 °C for 5 s and 60 °C for 30 s. After the reaction, the relative expression levels of the target genes were analyzed using the 2^⁻ΔΔCt^ method. A reference gene was used for normalization to compare the differences in the mRNA expression levels of the target genes between the different treatment and control groups.^37^ All RT-qPCR primer sequences are presented in Table S1.

### Western blotting

Next, 5637 and T24 cells in the exponential growth phase were transferred into centrifuge tubes according to their grouping. Radioimmunoprecipitation assay buffer (P0013B; Beijing Solarbio Science & Technology Co., Ltd.) was added, and the cells were incubated on ice for 30 min. Protein extracts were obtained from the supernatant. The protein concentration was determined using the BCA protein quantification kit (P0398L; Beijing Solarbio Science & Technology Co., Ltd.). Then, 30 μg of protein was loaded into each well of an electrophoresis tank. Electrophoresis was performed at 120 V for 90 min to separate the proteins under an electric field. Then, the proteins were transferred onto polyvinylidene difluoride membranes. The current was set at 260 mA for 1 h. The membranes were incubated with 5% skim milk powder at 25 °C for 2 h, followed by incubation with suitable primary antibodies overnight at 4 °C. The primary antibodies were diluted according to the manufacturer’s instructions. Then, the membranes were incubated with the corresponding secondary antibodies at 25 °C for 2 h. Bands were visualized using a chemiluminescence imager (A44241CFR; Thermo Fisher Scientific) after adding the ECL chemiluminescent substrate (P0018AS; Thermo Fisher Scientific), according to the manufacturer’s instructions. The grayscale values of the target protein bands were recorded and analyzed using the ImageJ 1.52a software. The relative target protein expression was normalized to the grayscale value of the internal reference protein, glyceraldehyde-3-phosphate dehydrogenase. All antibody information and dilution ratios are presented in Table S2.

### Measurement of extracellular acidification rate and oxygen consumption rate

The detection of cellular metabolic activity was accomplished using the Agilent Seahorse XFe96 Analyzer system. Plasmid transfection, siRNA-mediated knockdown, or JuA treatment was carried out according to the experimental design. On the day of detection, the culture medium in the cell culture plate was replaced with detection solution pre-prepared using XF DMEM Basal Medium (103575-100, Agilent, China). The culture plate, together with the sensor cartridge plate that had been hydrated and loaded with the Mito Stress Test Kit (103015-100, Agilent, China) and the Glycolysis Rate Assay Kit (103344-100, Agilent, China), was subsequently placed into the Seahorse XFe96 Analyzer. The instrument ran according to the preset program. The oxygen consumption rate (OCR) and extracellular acidification rate (ECAR) of each sample well were monitored in real-time and synchronously through the solid-state optical sensors, enabling dynamic analysis of cellular mitochondrial respiration and glycolysis functions.

### Statistical analyses

SPSS software (version 20.0) was used for data analysis. All the data are presented in the form of the mean ± the standard error of the mean (SEM). Each mean value was derived from a minimum of three independent experiments. One-way analysis of variance, followed by Tukey’s post-hoc test, was used for multigroup comparisons, and the *t*-test was used for two-group comparisons. Statistical significance was set at *P* < 0.05.

## Supplementary Material

Supplementary material.docx Supplementary material.docx

## Data Availability

The datasets used and/or analyzed during the current study are available from the corresponding author on reasonable request.
