# Supplementary material for: Jujuboside A induces bladder cancer cell apoptosis by inhibiting ATP1A2-mediated mitochondrial energy metabolism regulation
Source: Cancer Biol Ther. 2026 Jan 14;27(1):2615418. doi: 10.1080/15384047.2026.2615418 (PMC12818801; doi:10.1080/15384047.2026.2615418)
Supplement: Supplementary material.docx [file KCBT_A_2615418_SM8572.docx]

**Table S1 QPCR Primer sequences and the siRNA interference sequences and the ATP1A2 overexpression sequences**

| Gene | Forward Primer (5′–3′) | Reverse Primer (5′–3′) |
| --- | --- | --- |
| ATP1A2 | TCTATCCACGAGCGAGAAGAC | CCATGTAGGCATTTTGAAAGGC |
| GAPDH | GGAGCGAGATCCCTCCAAAAT | GGCTGTTGTCATACTTCTCATGG |
| Gene | SS Sequence | AS Sequence |
| SiNC | AAUUCUCCGAACGUGUCACGU | ACGUGACAGGUUCGGAGAAUU |
| SiATP1A2-1 | GCUCGACAAGGAGAUGCAAGA | UUGCAUCUCCUUGUCGAGCGG |
| SiATP1A2-2 | GAUUAGACACUAUGUGUUAGA | UAACACAUAGUGUCUAAUCUG |
| SiATP1A2-3 | GAGAGAAGAUGCAGAUCAACG | UUGAUCUGCAUCUUCUCUCCC |
| Gene | Primer sequence (5 '-3') | |
| pcDNA3.1-ATP1A2-F | TACCGAGCTCGGATCCGCCACCATGGGCCGTGGGGCTGGCCG | |
| pcDNA3.1-ATP1A2-R | GATATCTGCAGAATTCTCAGTAGTATGTCTCCTTCTC | |

**Table S2 Antibody information used in this study**

| Gene name | Manufacturer | Article number | Dilution ratio |
| --- | --- | --- | --- |
| ATP1A2 | Abcam | ab166888 | 1: 1000 |
| ATP5A | Abcam | ab110273 | 1: 1000 |
| COX1 | proteintech | 13393-1-AP | 1: 1000 |
| COX2 | proteintech | 27308-1-AP | 1: 1000 |
| NDUFA1 | Abclonal | A20940 | 1: 1000 |
| UQCRC2 | Affinity | DF12339 | 1: 1000 |
| MTCO1 | HUABIO | HA722838 | 1: 1000 |
| SDHB | Affinity | DF12732 | 1: 1000 |
| NDUFB8 | Affinity | DF9666 | 1: 1000 |
| Bcl-2 | Affinity | AF6139 | 1: 1000 |
| Bax | HUABIO | ER0907 | 1: 5000 |
| Caspase-3 | Abcam | ab32351 | 1: 5000 |
| Cleaved-Caspase-3 | Abcam | ab32042 | 1: 500 |
| GAPDH | proteintech | 60004-1-Ig | 1: 50000 |
| Goat Anti-Rabbit IgG H&L (HRP) | Affinity | S0001 | 1: 10000 |
| Goat Anti-Mouse IgG H&L (HRP) | Affinity | S0002 | 1: 10000 |
